# Molecular Markers Associated to Two Non-allelic Genic Male Sterility Genes in Peppers (*Capsicum annuum* L.)

**DOI:** 10.3389/fpls.2018.01343

**Published:** 2018-10-16

**Authors:** Ponnam Naresh, Shih-wen Lin, Chen-yu Lin, Yen-wei Wang, Roland Schafleitner, Andrzej Kilian, Sanjeet Kumar

**Affiliations:** ^1^Central Horticultural Experiment Station, ICAR-Indian Institute of Horticultural Research, Bhubaneswar, India; ^2^World Vegetable Center (WorldVeg), Tainan, Taiwan; ^3^Diversity Array Technologies, Canberra, ACT, Australia

**Keywords:** genic male sterility, genotyping by sequencing, hot pepper, hybrid seed production, SNP, sweet pepper

## Abstract

Male sterility is of high importance in hybrid seed production of hot and sweet peppers. Genic (or nuclear) male sterility (GMS) is a simply inherited (usually monogenic recessive) and highly stable trait. However, one major disadvantage of using GMS is 1:1 segregation of male sterile to male fertile plants in every subsequent generation. Molecular markers tightly linked to genic male sterility (*ms*) genes would facilitate an efficient and rapid transfer of *ms* genes into different genetic backgrounds through marker-assisted backcrossing. The two non-allelic genic male sterility genes *ms3* and *ms*_w_ in hot and sweet pepper backgrounds, respectively, are monogenic recessive. Genotyping by sequencing (GBS) in an F_2_ population segregating for *ms3* gene in hot pepper and in an F_6_ inbred near-isogenic line (NIL) population segregating for *ms*_w_ gene in sweet pepper yielded 9,713 and 7,453 single nucleotide polymorphism markers, respectively. Four candidate SNPs co-segregating with *ms3* gene and one co-segregating with *ms*_w_ gene were identified by bulk segregant analysis and physically mapped to chromosomes 1 and 5, respectively. In hot pepper, two markers [HPGMS2 (CAPS) and HPGMS3 (dCAPS)] located 3.83 cM away from the *ms3* gene and in sweet pepper the dCAPS marker SPGMS1 co-segregated (completely linked) with the *ms*_w_ gene were developed. These markers will increase the efficacy of the male sterility genes for pepper breeding, as they can be useful in developing the genic male sterile lines in parental inbred lines of commercial hybrids through marker-assisted backcrossing, hybrid seed production, and genetic purity testing of hybrid seeds.

## Introduction

The mechanism of male sterility has long been of interest among plant scientists and breeders. This interest stems from the use of male sterility systems to produce hybrids and their seeds in a cost-effective manner, as well as the differential/spatial expression of male sterility genes, the interaction between the nuclear and cytoplasmic genome, and serves as a model to better understand the endosymbiosis. Peppers (*Capsicum* spp.) are important commercial cash crops valued globally for their pungent (hot pepper) and non-pungent (sweet pepper) fruits that are used as a spice and vegetable, respectively. Both nuclear male sterility (genic male sterility, GMS) and cytoplasmic male sterility (CMS) systems are used to produce the cost-effective commercial pepper hybrid seed (Dhaliwal and Jindal, 2014; Lin et al., 2016). The CMS hot pepper lines have been used for commercial hybrid seed production, especially in India (Reddy et al., 2015; Lin et al., 2016; Schreinemachers et al., 2016). However, the use of the CMS system in sweet pepper has been limited because of high instability of male sterility expression at low temperatures and poor fertility restoration (*Rf*) ability in most of the sweet pepper genotypes (Lin et al., 2015). Since two independently isolated and commercially utilized S-cytoplasms of pepper are genetically similar (Kumar et al., 2009), the diversification of S (CMS)-cytoplasm is advisable to avoid risks associated with vulnerability of commercial hot pepper hybrids derived from single S-cytoplasm (Kumar et al., 2009; Yeh et al., 2016). To avoid such risks due to the monopolistic use of the same S-cytoplasm in peppers, GMS lines with highly stable and absolute expression of male sterility (Dhaliwal and Jindal, 2014) should also be increasingly used. More than 20 monogenic recessive nuclear male sterile (*ms*) genes in pepper are known (Dhaliwal and Jindal, 2014). The ethyl methanesulphonate (EMS)-induced *ms10* allele (originally named *ms509*) (Daskalov and Poulos, 1994) was found to be allelic with the natural *msk* and *ms2* alleles but was non-allelic to the *ms1* allele (Shifriss, 1997). Molecular markers linked to *ms1* (Lee et al., 2010a), *ms3* (Lee et al., 2010b), *ms8* (Bartoszewski et al., 2012), *ms10* (Aulakh et al., 2016), and to one *ms* with undisclosed or unknown origin (Lee et al., 2012) have been reported. The *ms8* and *ms10* genes were mapped to chromosome 4 and chromosome 1, respectively. The *ms3*-linked AFLP converted CAPS marker was reported, but the primer sequence and chromosome location were not provided (Lee et al., 2010b).

Genotyping by sequencing (GBS) is an efficient technology for the simultaneous discovery and genotyping of SNPs (Elshire et al., 2011; Taranto et al., 2016). Because of its cost effectiveness, GBS has been widely adopted for high density genotyping for linkage analyses, diversity studies, and genome wide association studies (He et al., 2014). The SNP markers have become a broadly used marker system due to the relative abundance of SNPs in most genomes and the co-dominant and bi-allelic nature of these markers (Mammadov et al., 2012). Once the SNP markers associated with a trait of interest are identified, for genotyping large numbers of individuals, suitable SNP assays can be developed (Semagn et al., 2014) or the SNPs can be converted to PCR-based CAPs or dCAPs markers (Lee et al., 2010b; Schafleitner et al., 2016).

The molecular markers linked to the recessive male sterility genes provide an unprecedented increase in efficacy for developing male sterile female inbred lines for hybrid seed production by identifying the male sterile and male fertile plants at the seedling stage. Hence, this study was conducted to perform an allelic test between two monogenic recessive genic male sterility genes and develop and validate molecular markers linked to male sterility genes using GBS. The results are presented and discussed in the regards to currently available molecular markers linked to *ms* genes in peppers for their wider application in pepper breeding.

## Materials and Methods

### Plant Materials

#### Male Sterile Lines and Genes

Two GMS lines, one each in the genetic background of hot and sweet peppers, were used. The hot pepper GMS line possessed the monogenic recessive *ms3* allele for male sterility. The *ms3* allele was originally derived from the hybrid “Novator F_1_,” which was provided to the World Vegetable Center (WorldVeg) by the Vegetable Crops Research Institute (VCRI), Hungary in 1987. For mapping the *ms3* gene and for marker development, 71 F_2_ plants derived from a cross between Novator (*ms3ms3*) and PBC315 (*Ms3Ms3*) were phenotyped and 47 of these were used for GBS. For *ms3*-associated molecular marker validation, a separate F_2_ population of 183 lines was used. The sweet pepper male sterile line possessed an unknown *ms* allele (designated temporarily as *ms*_w_) derived through continuous selfing and simple selection of a GMS-based sweet pepper hybrid “Forever” developed by Syngenta Private Limited. A stable F_6_ inbred population of 91 plants segregating into 50% male sterile (*ms*_w_*ms*_w_) and 50% male fertile (*Ms*_w_*ms*_w_) progenies was created and used for mapping and marker development.

#### Crosses for Testing Allelism of *ms* Genes

Since the sweet pepper male sterile line segregated into 50% male sterile (*ms*_w_*ms*_w_) and 50% male fertile (*Ms*_w_*ms*_w_) and the exact genotype of hot pepper male fertile plants was unknown at male sterility locus (*Ms3Ms3* or *Ms3ms3*), we crossed one hot pepper male sterile plant (*ms3ms3*) with one sweet pepper male fertile plant (*Ms*_w_*ms*_w_) for testing allelism between the *ms3* and the *ms*_w_ alleles. A total of 60 F_1_ plants were phenotyped for male sterility expression with the hypothesis that both the alleles would be allelic if F_1_ plants segregate at a 1:1 ratio of male sterility and male fertility.

### Phenotyping for Male Sterility

**FIGURE 1:**
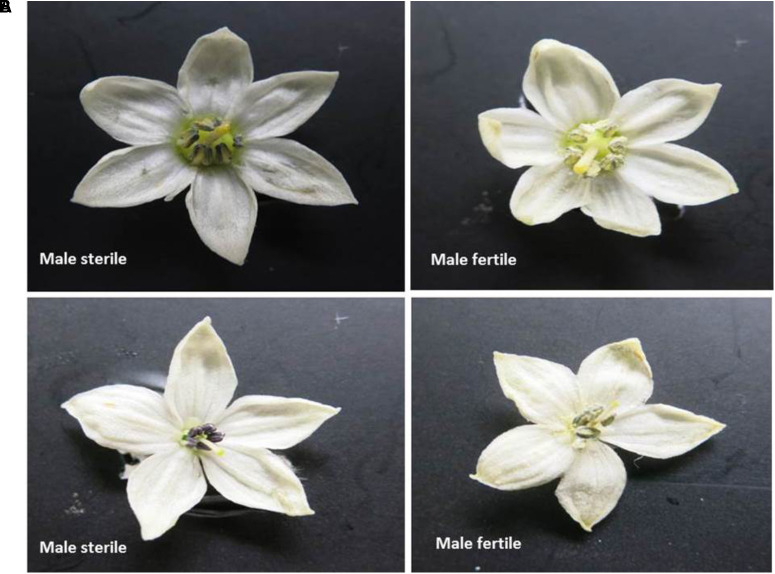
Flowers of male sterile and male fertile plants. **(A)** Sweet pepper, **(B)** Hot pepper.

**FIGURE 2:**
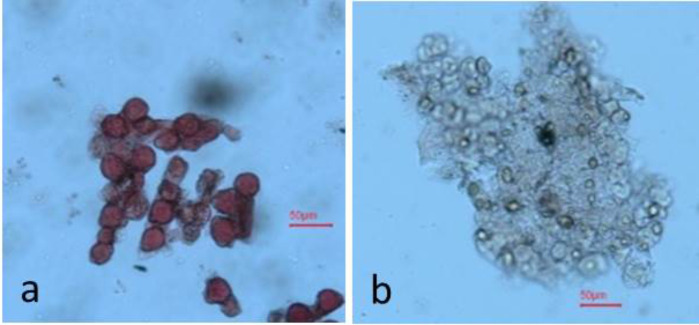
Pollen of **(a)** male fertile (stained), and **(b)** male sterile (unstained) plants.

Seeds were sown in small pots (6 inches diameter) in an insect-proof nethouse at WorldVeg, Tainan, Taiwan. All the seedlings were allowed to flower and set fruits. Ten freshly opened flowers of each plant were examined visually by physically tapping on the anthers of the flowers and observing the presence of abundant pollen as a powdery mass (male fertile) or absence of a powdery mass (male sterile) (**Figure 1**). Ambiguous flowers were tested for pollen viability by 1% acetocarmine staining (Marks, 1954; **Figure 2**). The final phenotype of each plant was assessed based on their ability (male fertile) or inability (male sterile) to produce fruits with seeds after self-pollination.

### Chi Square Test

The probability values between the expected and observed segregation ratios of male sterile and male fertile plants were tested through Chi square for goodness of fit in the Microsoft excel sheet. The calculated chi square value was compared with the *t*-value. If the calculated value was found to be lower than the *t*-value, it was considered non-significant, indicating the best fit for the observed and expected ratios.

### Genotyping by Sequencing

From 71 F_2_ hot pepper plants, 47 plants consisting of 28 male fertile (*Ms3Ms3* or *Ms3ms3*) and 19 male sterile (*ms3ms3*) were selected for GBS. In the case of sweet pepper, from 91 NILs, 47 plants consisting of 23 male fertile (*Ms*_w_*ms*_w_) and 24 male sterile (*ms*_w_*ms*_w_) were selected for GBS. The DNA was isolated using the DNEasy Mini Kit (Qiagen) according to the instructions of the manufacturer. Fluorometric quantification of the DNA was done on a Qubit device (Invitrogen). Sequencing of both the sweet and hot peppers was done at Diversity Array technologies, Canberra, Australia using the DArTSeq^TM^ technology (Sansaloni et al., 2011). Restriction enzymes *PstI* and *MseI* were used for complexity reduction, and digestion/ligation reactions were performed as described by Kilian et al. (2012), replacing a single *PstI* compatible adapter with a *PstI* barcode adapter, also including the Illumina flow cell attachment and sequencing primer sequence, and a *MseI* common adapter. Barcodes were used as described (Elshire et al., 2011). For PCR amplification, primer pairs that only bound to fragments containing the *PstI* adapter on one side and the *MseI* adapter on the other side were used, thereby leading to selective amplification of fragments having both restriction enzyme cutting ends. The PCR conditions were 1 min at 94°C for initial denaturation; 30 cycles each consisting of 20 s at 94°C for denaturation, 30 s at 58°C for annealing, and 45 s at 72°C for extension; and a final extension step for 7 min at 72°C. After PCR, equimolar amounts of the amplification products from each sample were bulked and applied to c-Bot (Illumina) bridge PCR followed by sequencing on Illumina Hiseq 2500 (Ren et al., 2015). Sequences generated from each lane were processed using the proprietary DArT analytical pipelines, as described (Ren et al., 2015).

### Bulk Segregant Analysis

In total, 9713 and 7453 SNP markers were used for bulk segregant analysis for the hot and sweet pepper populations, respectively. Individual male fertile and male sterile plants used for genotyping were grouped into male fertile and male sterile bulks, respectively. In hot pepper, the F_2_ population segregation did not deviate from a 1:2:1 ratio for the *ms3* gene (*Ms3Ms3*: *Ms3ms3*: *ms3ms3)*, therefore heterozygous or homozygous SNPs (N/N or N/n) in male fertile plants (28 plants) and homozygous SNPS (n/n) in male sterile plants (19 plants) were selected as candidates associated with the *ms3* gene. For the sweet pepper NIL population, segregation did not deviate from a 1:1 ratio into heterozygote male fertile (*Ms*_w_*ms*_w_) and homozygote recessive male sterile (*ms*_w_*ms*_w_) plants, and hence a heterozygous SNP (N/n) in male fertile plants (23 plants) and homozygous (n/n) in male sterile plants (23 out of 24 male sterile plants) were selected as candidate SNPs for marker development.

### Marker Development

The sequence context of the candidate SNPs was searched in the *Capsicum annuum* cv CM334 genome sequence (Kim et al., 2014) by BLAST alignment^1^ to acquire elongated sequences for the marker development. The SNPs were converted to cleaved amplified polymorphic sequences (CAPS) using the CAPS designer tool ^2^. In cases where conversion of SNPs to CAPS was not possible, dCAPS markers were developed using the dCAPS finder^3^ (Neff et al., 1998). Primer 3 input (version 0.4.0) program^4^ was used for designing primers.

### Marker Validation

Genomic DNA was extracted from actively growing young leaves (Fulton et al., 1995). Polymerase chain reaction was performed using specific primers listed in **Table 2**. Each PCR contained 30 ng of DNA, 0.2 mM of each primer, of deoxy ribonucleotides, 50 mM KCl, 10 mM TrisHCl (pH 8.3), 1.5 mM MgCl2, and 0.5 unit of Taq DNA polymerase. The PCR conditions include initial denaturation at 94°C for 5 min, followed by 30 cycles of 94°C for 30 s, annealing temperature for 45 s, 72°C for 45 s, and final extension at 72°C for 7 min. The annealing temperature was optimized for each primer combination by gradient PCR.

## Results

### Phenotypic Segregation

Seventy-one and 183 F_2_ plants were, respectively, used for molecular marker development and validation of hot pepper segregating for *ms3* gene. Ninety-one plants of the sweet pepper NIL population segregating for the unknown *ms* gene (*Ms*_w_*ms*_w_/*ms*_w_*ms*_w_) were phenotyped for the expression of male sterility.

In the first set of the F_2_ population of hot pepper, 52 out of 71 plants were male fertile and 19 were male sterile, which were in agreement with the single recessive gene ratio of 3:1 (χ^2^ = 0.089 at *P* = 0.76). Likewise, in the second set of 183 F_2_ plants, the segregation ratio was also found to be a good fit to a single recessive gene segregation (χ^2^ = 0.117 at *P* = 0.73) (**Table 1**). The segregation of the NIL population of sweet pepper did not deviate from a 1:1 ratio for male fertile: male sterile, which fit well in the expected single gene segregation (χ^2^ = 0.01 at *P* = 0.92) (**Table 1**). Segregation ratios revealed that the male sterility genes in both populations of sweet pepper (*ms*_w_) and hot pepper (*ms3*) are monogenic recessive.

**Table 1:** Segregation of male fertile and male sterile phenotypes in F_2_ population of hot pepper and near isogenic lines population of sweet pepper.

Population	Total number of plants	Phenotype observed (plants)		Expected ratio	*χ* ^2^	*P*-value at *P* ≤ 0.05
		Male fertile	Male sterile			
**Hot pepper F_2_ population derived from the cross Novator × PBC315**						
First set	71	52	19	3:1	0.089	0.76
Second set	183	139	44	3:1	0.117	0.73
**Sweet pepper stable F_6_NIL (*ms_w_ms_w_* × *Ms_w_ms_w_*) population derived from Forever hybrid**						
NIL population	91	46	45	1:1	0.01	0.92

### Allelism Test

Sixty F_1_ plants produced from a cross between male sterile hot pepper (*ms3ms3*) and the sweet pepper plants with the unknown heterozygous male sterility allele (*Ms*_w_*ms*_w_) were found to be 100% male fertile, indicating that these two genes (*ms3* and *ms*_w_) are non-allelic. Alternatively, had these been different alleles of the same gene there would have been 1:1 segregation of male fertile and male sterile plants.

### Genotyping by Sequencing

From the 9,713 SNPs identified in the hot pepper library, 6909 (71%) could be mapped to the reference genome. Likewise, in the sweet pepper library, 7453 SNPs were identified and the proportion of mapped markers was 68.4%, which is similar to the hot pepper library. The SNPs covered all the 12 chromosomes of pepper, with 458 to 707 SNPs per chromosome for hot pepper and 376–533 SNPs per chromosome for sweet pepper. From all the SNPs, 2,804 and 2,354 SNPs could not be attributed to a chromosome in hot and sweet peppers, respectively. The average distance between two markers in hot pepper amounted to 497 kb (maximum distance 15 Mb) while it was 656 kb (maximum distance 16 Mb) in sweet pepper.

### Bulk Segregant Analysis

Since the expression of male sterile genes *ms3* and *ms*_w_ was strictly monogenic recessive, we conducted a bulk segregant analysis (BSA) comparing the marker genotypes between bulks. For hot pepper, 4 SNPs were selected as candidates for molecular markers from 47 genotyped plants because they were homozygous recessive (n/n) in 19 male sterile (*ms3ms3*) plants, heterozygous (N/n) in 20 male fertile (*Ms3ms3*) plants, and homozygous dominant (N/N) in 8 male fertile (*Ms3Ms3*) plants. All these four candidate SNPs were physically mapped on chromosome 1 at positions 70,730,824–96,273,123 bp and co-segregated with male sterility.

In sweet pepper, only one SNP marker was heterozygous in all 23 male fertile (*Ms*_w_*ms*_w_) plants and homozygous recessive in 23 out of 24 male sterile plants (*ms*_w_*ms*_w_). This candidate SNP was physically mapped to chromosome 5 at the physical position 32,890,811.

### Marker Development

The candidate SNPs were converted to PCR-based CAPS and dCAPS markers. In hot pepper, for candidate SNPs 1 and 3 CAPS markers were developed, whereas for candidate SNPs 2 and 4, dCAPS markers were developed because these SNPs could not be converted to CAPS markers (SNP accession # PRJEB28302). In sweet pepper, the candidate marker was physically converted to a dCAPS marker. The details of the (d)CAPS markers such as primer sequence, restriction enzyme, and PCR product sizes are presented in **Table 2**.

**Table 2:** Primer sequences and amplification profile of markers for *ms3* and *ms*_w_ genes in peppers.

Name & marker type	Chromosome: position	Forward primer	Reverse primer	Enzyme	Annealing temperature	Amplified allele size (bp)		
						Homozygous dominant	Heterozygous	Homozygous recessive
**Markers for *ms3* gene**								
HPGMS2CAPs	1:96273123	GGTACTTTGA CCCTCATAA TTGG	TTGTTTGT GGTGTACG TGCT	Hpy188I	63.0°C	140bp	140bp, 93bp, 47bp	93bp, 47bp
HPGMS3dCAPs	1:71574503	GGGATGTTCA GTCTCATGT	GACTTTTTCC CGATCTCGG	Fok1	46.3°C	93bp, 35bp	128bp, 93bp, 35bp	128bp
**Marker for *ms*_w_ gene**								
SPGMS1dCAPs	5:32890811	GTAGTGATTGG TATGTCCA	CGTAAGT AGAAGCTT ATGA	HinfI	50°C	-	132bp, 111bp, 21bp	111bp, 21bp

### Validation of Markers and Mapping

Molecular markers were validated in sets of phenotyped plants.

**FIGURE 3:**
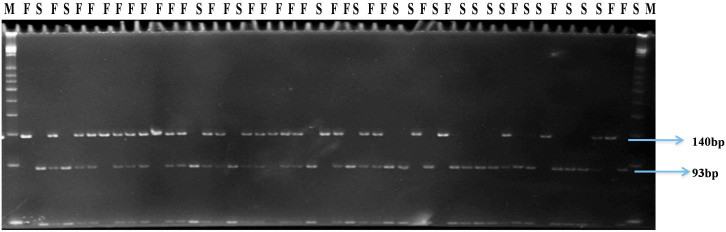
Gel profile of HPGMS2-CAPS marker linked to *ms3* gene in an F_2_ segregating population; M, 50 bp DNA ladder; F, male fertile (*Ms3Ms3*/*Ms3ms3*); S, male sterile (*ms3ms3*).

**FIGURE 4:**
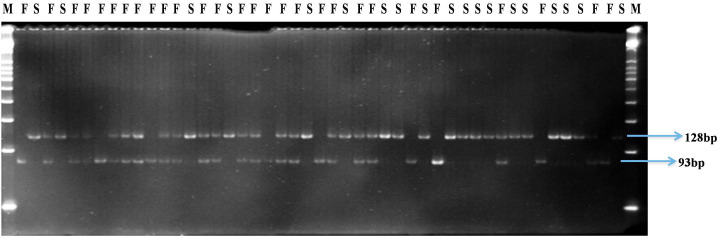
Gel profile of HPGMS3-dCAPS marker linked to *ms3* gene in an F_2_ segregating population; M, 50 bp DNA ladder; F, male fertile (*Ms3Ms3*/*Ms3ms3*); S, male sterile (*ms3ms3*).

Four SNP markers physically mapped on chromosome 1 at positions 71,091,336 (SNP1), 96,273,123 (SNP 2), 71,574,503 (SNP 3), and 70,730,824 (SNP 4) co-segregated with the male sterile/fertile phenotype. In the initial validation of the GBS data in the 47 genotyped plants of the BSA, HPGMS 1 primers designed for SNP 1 was not polymorphic, indicating that the GBS gave a false positive SNP at this position. In contrast, HPGMS2 and HPGMS3 were polymorphic among male fertile and sterile plants and co-segregated perfectly with the male sterile/fertile phenotype in the 47 genotypes. The HPGMS4 marker showed partial digestion interfering with scoring and was discarded. Therefore, the HPGMS2 (CAPS) and HPGMS3 (dCAPS) markers were validated in a test set of 183 F_2_ plants with known male fertility phenotype. Both markers differentiated heterozygous (*Ms3ms*3- male fertile), homozygous dominant (*Ms3Ms3*–male fertile), and homozygous recessive (*ms3ms3* – male sterile) genotypes without any ambiguity (**Table 3** and **Figures 3**, **4**). Seven recombinants were found in 183 test plants between HPGMS2 and HPGMS3, and the sterility locus suggested a genetic distance between both markers and the *ms3* gene to be 3.83 cM.

**Table 3:** Segregation of SNP-derived markers linked to *ms3* and *ms*_w_ genes in hot pepper (F_2_) and sweet pepper (near isogenic; NIL) populations.

Marker	Population	Total number of plants	Genotype observed (no of plants)			Expected ratio	*χ* ^2^	*P*-value at *P* ≤ 0.05
			Male fertile (homozygous dominant)	Male fertile (heterozygous)	Male sterile (homozygous recessive)			
**Hot pepper F_2_ population (*ms3*) derived from the cross Novator × PBC 315**								
HPGMS2	First set for marker development	47	9	19	19	Co-segregated with male sterile/fertile plants and validated the GBS data		
	Second set for marker validation	183	46	86	51	1:2:1	0.93	0.63
HPGMS3	First set for marker development	47	9	19	19	Co-segregated with male sterile/fertile plants and validated the GBS data		
	Second set for marker validation	183	45	87	51	1:2:1	0.83	0.66
**Sweet pepper stable F_6_ inbred NIL population (*ms_w_ms_w_* × *Ms_w_ms_w_*) derived from Forever hybrid**								
SPGMS1	NIL	91	-	46	45	1:1	0.01	0.92

**FIGURE 5:**
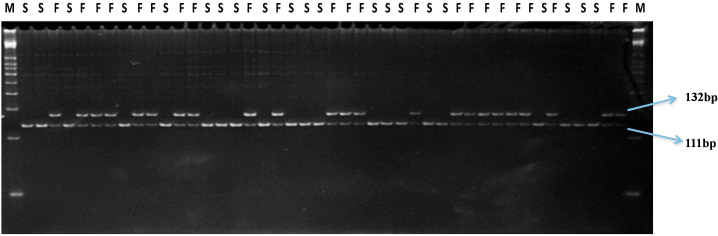
Gel profile of SPGMS1-dCAPS marker linked to *ms*_w_ gene in an F_6_ NIL population; M, 50 bp DNA ladder; F, male fertile (*Ms*_w_*ms*_w_); S, male sterile (*ms*_w_*ms*_w_).

In sweet pepper, the dCAPS marker SPGMS1 (**Table 3**) co-segregated with sterility and differentiated the heterozygous (*Ms*_w_*ms*_w_) male fertile plants from the homozygous recessive (*ms*_w_*ms*_w_) male sterile plants (χ^2^ = 0.01 at *P* = 0.92) (**Table 3** and **Figure 5**).

## Discussion

Hybrid seed production through manual emasculation and pollination in pepper is labor demanding and not cost-effective. The introduction of male sterility systems can reduce up to 40% of the labor costs by eliminating the manual emasculation process (Lin et al., 2015). Both GMS and CMS systems are available and well documented in peppers. For commercial hybrid development and seed production, the CMS is currently used only in hot pepper, while the GMS is used in both hot and sweet peppers (Dhaliwal and Jindal, 2014). This is because fertility restoration in sweet pepper is weak (Lin et al., 2015) and the expression of male sterility is conditioned by the presence of several *restorer-of-fertility* (*Rf*) loci including partial restoration loci in pepper (Lee et al., 2008). However, the worldwide commercially used male sterile cytoplasm is genetically similar (Kumar et al., 2009). This monopolistic use of a single source of a male sterile cytoplasm is not desirable and needs either diversification of CMS source in the long term, or promotion of GMS-based hybrid crops in the short term. Hence, to increase the efficacy of GMS in hybrid seed production in hot and sweet peppers, we mapped the *ms3* and *ms*_w_ genes and developed molecular markers to distinguish the alleles for male fertility and sterility. Both genes (*ms3* and *ms*_w_) caused complete male sterility and their inheritance was monogenic recessive. Among the approximately 20 known spontaneously occurring or artificially induced nuclear male sterile genes in peppers (Wang and Bosland, 2006; Dhaliwal and Jindal, 2014), all are monogenic recessive (Shifriss, 1997; Lee et al., 2012; Aulakh et al., 2016) with the single exception of the dominant mutant male sterility gene *Dms* (Daskalov and Poulos, 1994). Very few allelic tests have been performed among these male sterile genes (Dhaliwal and Jindal, 2014). However, *ms10* was found to be non-allelic to *ms3* (Yu, 1990) but allelic to the *msk* gene (Yu, 1985). We found that *ms3* and *ms*_w_ are non-allelic as all the F_1_ plants from the hybridization *ms3ms3* ×*Ms*_w_*ms*_w_ were male fertile. This non-allelic relationship established through a genetic test was also confirmed through genotypic sequence data, as *ms3* and *ms*_w_ genes were physically mapped on different chromosomes, chromosomes 1 and 5, respectively (**Table 4**). The *ms10* (originally *ms509*) gene has also been recently mapped on chromosome 1 and the associated Simple Sequence Repeat (SSR) marker (AVRDC-PP12) was developed (Aulakh et al., 2016). However, as expected, the AVRDC-PP12 marker for *ms10* did not produce polymorphism in our *ms3* population (data not shown), ascertaining that both are non-allelic. GBS revealed to be highly efficient in obtaining markers tightly linked to monogenic male sterility (*ms*) genes using a small population. Hence, the method is rapid and involves relatively low cost (He et al., 2014). We employed GBS to identify the SNP markers for *ms3* and *ms*_w_ genes and successfully converted the SNP markers into PCR-based CAPS and dCAPS markers and validated these markers in segregating populations. Since both the male sterile genes were monogenic recessive, 47 plants were sufficient to identify the markers associated with *ms*_w_ in the NIL population and *ms3* in the F_2_ population. For the *ms3* gene, one CAPS (HPGMS2) and one dCAPS (HPGMS3) marker were developed. Both of these markers co-segregated with the *ms3* gene and the phenotypes in 176 out of 183 plants (**Table 3**), and therefore are suggested to be located at 3.82 cM from the *ms3* gene. In spite of the distance between the markers and the *ms3* gene, these markers could be used in marker-assisted selection (MAS) and hybrid seed production by facilitating identification of most of the male sterile and male fertile plants at the seedling stage. As expected, six successive generations of backcrossing introgression carrying the male sterility gene in the NIL population was small, and therefore, only one candidate marker was obtained for this population, while four candidates were obtained for the F_2_ population. The dCAPS marker SPGMS1 was developed for the *ms*_w_ gene for sweet pepper, which showed complete co-segregation with the *ms*_w_ gene differentiating the heterozygous male fertile plants from the homozygous recessive sterile plants. MAS is important for the GMS system because (i) transferring the *ms* gene into different genetic backgrounds through conventional backcrossing is tedious and time-consuming, as selfing after each successive backcross is required to select the male fertile heterogonous plant and (ii) for hybrid seed production, 50% male fertile plants need to be identified and removed in the hybrid seed production plot, which is also a time- and resource-consuming process. Both these obstacles are overcome by the use of molecular markers linked to the male sterility locus. Hence, the co-dominant molecular markers linked to the *ms3* (HPGMS2 and HPGMS3) and *ms*_w_ (SPGMS1) genes developed in this study will greatly reduce the amount of time and labor resources required for early (seedling stage) identification of the heterozygous male fertile plants (*Msms*) in backcross breeding (Dhaliwal and Jindal, 2014). These molecular markers will be equally useful in selecting the male sterile seedlings for hybrid seed production (Lee et al., 2010b) and also in hybrid purity testing (Jang et al., 2004). In addition, the molecular markers identified in this study may be useful in the cloning of *ms3* and *ms*_w_ genes for various uses in strategic and applied research including map-based cloning of these two male sterility genes.

**Table 4:** Markers reported for non-allelic nuclear male sterile genes in pepper.

*ms* gene	Marker type	Chromosome location	Reference
*ms1*	AFLP	Not reported (sequence not available)	Lee et al., 2010a
*ms1*	HRM	Chromosome 5	Jeong et al., 2018
*ms3*	AFLP- CAPS	Not reported (sequence not available)	Lee et al., 2010b
*ms3*	SNP	Chromosome 1	This study
*ms8*	SCAR	Chromosome 4	Bartoszewski et al., 2012
*ms10*	SSR	Chromosome 1	Aulakh et al., 2016
*ms*_w_	SNP	Chromosome 5	This study

In peppers, although more than 20 *ms* genes have been reported, molecular markers linked to male sterility loci are known for only a few of them (**Table 4**). AFLP markers associated with the *ms1* gene (Lee et al., 2010a), CAPS markers associated with the unknown *ms* gene (Lee et al., 2010c), SCAR markers associated with the *ms8* gene (Bartoszewski et al., 2012), and a distantly linked SSR marker for the *ms10* gene (Aulakh et al., 2016) have been developed. However, for the *ms3* gene, the marker information (primer sequence) has not previously been revealed (Lee et al., 2010b; **Table 4**) and was not available for public use. For the first time we are reporting markers for the *ms3* gene with complete and detailed information for public use. During the preparation of this manuscript, Jeong et al. (2018) reported that *ms1* in Magnipio hybrid of Syngenta is located on chromosome 5, similar to *ms*_w_ derived from Forever hybrid of Syngenta mapped in this study (**Table 4**). Moreover, the location of the marker for *ms*_w_ (32187928) is within the fine mapped 869.9 kb *ms1* gene (31516016–32385930) reported by Jeong et al. (2018). Hence, it is highly likely that *ms*_w_ and *ms1* are the same gene (allelic); however, a genetic test between *ms*_w_ and *ms1* would be desirable to confirm their functional allelism.

Nuclear male sterile genes in peppers are usually highly stable and possess a monogenic simply inherited trait; therefore, they could be relatively easy to use in breeding programs if molecular markers are available. We employed GBS technology to develop molecular markers linked to two non-allelic male sterility genes (*ms3* and *ms*_w_) in peppers. These markers will facilitate rapid and cost-effective development of GMS lines, useful in hybrid seed production and genetic purity testing of hybrid seeds in peppers.

## Author Contributions

PN executed the research work and data analysis and led the manuscript preparation. SWL helped in greenhouse experiments. CYL helped in marker development. YWW helped in markers validation. RS supervised the genotyping by sequencing progress and revised the manuscript. AK helped in GBS data analysis. SK planned the entire work and helped substantially to develop the draft and revisions of the manuscript.

## Conflict of Interest Statement

The authors declare that the research was conducted in the absence of any commercial or financial relationships that could be construed as a potential conflict of interest.

## Footnotes

1
https://www.solgenomics.net/tools

2
https://solgenomics.net/tools/caps_designer/caps_input.pl

3
http://helix.wustl.edu/dcaps/dcaps.html

4
http://bioinfo.ut.ee/primer3-0.4.0
