# Corrigendum: Molecular markers associated to two non-allelic genic male sterility genes in peppers (*Capsicum annuum* L.)

**DOI:** 10.3389/fpls.2023.1288072

**Published:** 2023-10-12

**Authors:** Ponnam Naresh, Shih-wen Lin, Chen-yu Lin, Yen-wei Wang, Roland Schafleitner, Andrzej Kilian, Sanjeet Kumar

**Affiliations:** ^1^Central Horticultural Experiment Station, ICAR-Indian Institute of Horticultural Research, Bhubaneswar, India; ^2^World Vegetable Center (WorldVeg), Tainan, Taiwan; ^3^Diversity Array Technologies, Canberra, ACT, Australia

**Keywords:** genic male sterility, genotyping by sequencing, hot pepper, hybrid seed production, SNP, sweet pepper

In the published article, there was an error in **Table 2** Primer sequences and amplification profile of markers for *ms3* and *ms_w_* genes in peppers as published. The sequence (forward and reverse primer) of marker SPGMS1DCAPS was incorrectly published. The corrected **Table 2** Primer sequences and amplification profile of markers for *ms3* and *ms_w_* genes in peppers appears below.

**Table 2:** Primer sequences and amplification profile of markers for *ms3* and *ms_w_* genes in peppers.

Name & Marker type	Chromosome; position	Forward primer	Reverse primer	Enzyme	Annealing temperature	Amplified allele size (bp)		
						Homozygous dominant	Heterozygous	Homozygous recessive
Markers for *ms3* gene								
HPGMS2CAPs	1:96273123	GGTACTTTGACCCTCATAATTGG	TTGTTTGTGGTGTACGTGCT	Hpy188I	63.0°C	140bp	140bp, 93bp, 47bp	93bp, 47bp
HPGMS3dCAPs	1:71574503	GGGATGTTCAGTCTCATGT	GACTTTTTCCCGATCTCGG	Fok1	46.3°C	93bp, 35bp	128bp, 93bp, 35bp	128bp
Marker for *ms_w_* gene								
SPGMS1dCAPs	5:32890811	GTAGTGATTGGTATGTCCA	CGTAAGTAGAAGCTTATGA	HinfI	50°C	–	132bp,111bp, 21bp	111bp, 21bp

The authors apologize for this error and state that this does not change the scientific conclusions of the article in any way. The original article has been updated.

